# Angiopoietin-like protein 3 (ANGPTL3) deficiency and familial combined hypolipidemia

**DOI:** 10.7555/JBR.32.20170114

**Published:** 2019

**Authors:** Patrizia Tarugi, Stefano Bertolini, Sebastiano Calandra

**Affiliations:** 1. Department of Life Sciences, University of Modena and Reggio Emilia, Modena 41125, Italy; 2. Department of Internal Medicine, University of Genova, Genova 16148, Italy; 3. Department of Biomedical, Metabolic and Neural Sciences, University of Modena and Reggio Emilia, Modena 41125, Italy.

**Keywords:** angiopoietin-like protein 3, ANGPTL3 deficiency, loss of function variants, FHBL2

## Abstract

Three members of the angiopoietin-like (ANGPTL) protein family-ANGPTL3, ANGPTL4 and ANGPTL8- are important regulators of plasma lipoproteins. They inhibit the enzyme lipoprotein lipase, which plays a key role in the intravascular lipolysis of triglycerides present in some lipoprotein classes. This review focuses on the role of ANGPTL3 as emerged from the study of genetic variants of *Angptl3* gene in mice and humans. Both loss of function genetic variants and inactivation of *Angptl3* gene in mice are associated with a marked reduction of plasma levels of triglyceride and cholesterol and an increased activity of lipoprotein lipase and endothelial lipase. In humans with ANGPTL3 deficiency, caused by homozygous loss of function (LOF) variants of *Angptl3* gene, the levels of all plasma lipoproteins are greatly reduced. This plasma lipid disorder referred to as familial combined hypolipidemia (FHBL2) does not appear to be associated with distinct pathological manifestations. Heterozygous carriers of LOF variants have reduced plasma levels of total cholesterol and triglycerides and are at lower risk of developing atherosclerotic cardiovascular disease, as compared to non-carriers. These observations have paved the way to the development of strategies to reduce the plasma level of atherogenic lipoproteins in man by the inactivation of ANGPTL3, using either a specific monoclonal antibody or anti-sense oligonucleotides.

## Introduction

Angiopoietin-like proteins (ANGPTLs) represent a family of eight secreted glycoproteins that show structural homology to angiopoietins and carry distinct physiological functions, including putative roles in lipid metabolism, expansion of stem cells, inflammation, tissue remodeling and angiogenesis^[1^–^2]^. In recent years, three ANGPTLs, ANGPTL3, ANGPTL4 and ANGP-TL8, have been shown to play a role in lipid metabolism and in the regulation of plasma lipid levels^[3^–^4]^.

More specifically, ANGPTL 3, ANGPTL4 and ANG-PTL8 share a common feature, being, to a variable extent, negative regulators of the activity of lipoprotein lipase (LPL), the key enzyme involved in the intravascular lipolysis of triglyceride (TG) present in some lipoprotein classes, such as chylomicrons and very low density lipoproteins (VLDL)^[5^–^6]^.

This overview focuses on the role of ANGPTL3 in lipoprotein metabolism and the effect of its deficiency/inactivation in humans and animal models.

## ANGPTL3 deficiency: discovery of monogenic combined hypolipidemia in mice

The link between ANGPTL3 and plasma lipoprotein metabolism emerged from the identification of the KK/San mouse strain, a mutant strain derived from a colony of KK mice characterized by diabetes, obesity and hypertriglyceridemia. The KK/San mice, despite maintaining the phenotype of obesity and diabetes, had a marked decrease of plasma TG (>90%), as compared with the colony of KK mice. This severe hypotriglyceridemia was due to a marked reduction of plasma VLDL. These mice were found to be homozygous for a loss-of-function (LOF) variant of the *Angptl3* gene, causing the formation of a truncated Angptl3, and leading to a complete Angptl3 deficiency^[7]^. The hypolipidemia observed in this mutant mouse strain was found to be associated with an increased activity of LPL^[8]^. Since this observation, other studies conducted in *Angptl3 *^*-/-*^ mice not only confirmed that Angptl3 is an inhibitor of LPL, but also showed that complete Angptl3 deficiency is associated with a reduction of TG-containing lipoproteins (VLDL) and also with a reduction of cholesterol-carrying lipoproteins, such as LDL and HDL^[9]^. On the other hand, overexpression of Angptl3 in mice increased plasma TG by inhibiting the activity of LPL^[10]^.

## Familial combined hypolipidemia in humans

In humans, a lipoprotein phenotype similar to that observed in KK/San mice was firstly described by Musunuru *et al*. in a large family originally thought to be affected by familial hypobetalipoproteinemia (FHBL1) (OMIM#615558), in view of the low levels of plasma LDL-C^[11]^. Two siblings of this family showed extremely low levels of total cholesterol (TC) and LDL-C and low levels of TG and HDL-C. Exome sequencing revealed that these siblings were compound heterozygous for two LOF variants of ANGPTL3 [p.(E129*)/p.(S17*)], expected to cause a complete deficiency of ANGPTL3. This novel lipoprotein phenotype was designated Familial Combined Hypolipidemia (FHBL2, OMIM#605019)^[11]^. The genetic screening in the family led to the identification of 13 heterozygotes. These individuals showed intermediate plasma levels of LDL-C and TG between compound heterozygous and non-carrier family members with a gene-dosage effect. This was not the case for plasma HDL-C levels that were similar in heterozygous carriers and in non-carriers. The nonsense variant p.(S17*) of ANGPTL3 was also found in a cohort of hypolipidemic individuals living in a district of Central Italy, where the resequencing of *Angptl3* gene in nine families and in a large cohort of individuals of the local population (352 individuals) led to the identification of 62 carriers of this variant (8 homozygotes and 54 heterozygotes)^[12]^. In this survey homozygotes had undetectable plasma levels of ANGPTL3, low TG and cholesterol levels and a striking reduction of all lipoprotein classes (VLDL, LDL and HDL*)*; heterozygotes had a 50% reduction in circulating ANGPTL3 and reduced levels of TC and HDL-C, as compared to non-carriers and levels of LDL-C and TG similar to controls^[12]^. Furthermore, p.(S17*) homozygotes had significantly higher LPL activity and mass as compared to controls and lower plasma level of free fatty acids, which suggested a reduced lipolysis in adipose tissue^[13]^. Other individuals with familial combined hypolipidemia due to different LOF variants of *Angptl3* gene were identified in Italian and Spanish families, as well as in a cohort of subjects with primary hypocholester-olemia^[14^–^16]^. In a pooled analysis of carriers of LOF variants of *Angptl3* gene, Minicocci *et al*. evaluated the biochemical characteristics of 115 individuals carrying different LOF variants of *Angptl3* (including 14 homozygotes, 8 compound heterozygotes and 94 heterozygotes)^[17]^. These investigators showed that, as compared to controls, the carriers of two LOF alleles (homozygotes/compound heterozygotes) as well as carriers of a single LOF allele (simple heterozygotes) showed a significant reduction of all plasma lipoproteins.

From a clinical point of view, carriers of two *Angptl3* LOF alleles identified so far in family studies did not show a distinct pathological phenotype. More specifically, they did not show clinical manifestations of premature atherosclerosis or increased risk of ischemic heart disease, which might have been expected in view of the lifelong exposure to low levels of HDL-C (a known clinical predictor of risk of atherosclerotic cardiovascular disease)^[18]^. Minicocci *et al*. evaluated the vascular status in a group of FHBL2 subjects (7 homozygotes and 59 heterozygotes) carrying the *Angptl3* LOF mutation p.(S17*)^[19]^. They found that FHBL2 individuals did not show significant changes in carotid intima-media thickness (a surrogate marker for atherosclerosis) with respect to controls. These observations suggest that, despite the presence of low HDL-C levels, FHBL2 subjects are protected from developing premature atherosclerosis by the concomitant reduction of the levels of atherogenic lipoproteins such as VLDL and LDL.

Another key issue regarding the clinical phenotype in FHBL2 concerns the presence of fatty liver disease, a condition frequently encountered in individuals with FHBL1. The latter individuals, who have persistently low levels of TG and LDL-C, resulting from *APOB* gene LOF variants which impair the hepatic secretion of VLDL, usually develop fatty liver of variable severity^[20]^. Carefully conducted clinical studies have shown that in FHBL2 there was no increased prevalence of fatty liver or chronic liver disease with respect to controls^[21]^.

## LOF variants of *Angptl3* gene identified in population studies

Early genome wide association studies (GWAS) had shown that common and rare variants of *Angptl3* gene were associated with variations in plasma levels of TG, TC, LDL-C and HDL-C^[22^–^23]^. In addition, resequencing of *Angptl3* in some population studies had shown that some LOF variants were associated with reduced levels of plasma TG^[24]^. By sequencing the *Angptl3* gene in the DiscovEHR study participants and in four other population cohorts, Dewey *et al*. recently identified 400 subjects heterozygous for 13 different LOF *Angptl3* variants, with an estimated allele frequency of 1 in 237^[25]^. These heterozygous carriers had a significant reduction of TG, LDL-C and HDL-C levels and a 50% reduction of circulating ANGPTL3 as compared to non-carriers. In addition, the LOF variants of *Angptl3* were found to be associated with a 39% reduction of coronary artery disease (CAD)^[25]^.

In another recent study, *Angptl3* sequence data from case-control studies and a population-based cohort study led to the identification of 23 LOF variants^[26]^. These LOF variants were present in 130 of 40,112 participants (1 in 309 individuals). Furthermore, in heterozygous carriers of an *Angptl3* LOF variant, selected from a cohort of more than 20,000 individuals of the Myocardial Infarction Genetics Consortium studies, the plasma levels of TC, LDL-C and TG were reduced by 11%, 12% and 17%, respectively, with no significant changes in HDL-C as compared to non-carriers. In addition, the authors determined the relationship between *Angptl3* LOF variants and the risk of CAD. They found that heterozygous carriers of LOF variants in *Angptl3* had a 34% decreased risk of CAD. This reduced CAD risk was associated with lower levels of circulating ANGPTL3^[26]^. Collectively, these two large surveys strongly indicate that partial ANG-PTL3 deficiency due to heterozygosity for LOF *Angptl3* variants results in a reduced risk of CAD as compared to non-carriers, suggesting that ANGPTL3 may be a novel target to reduce the level of atherogenic lipoproteins^[25^–^26]^. An updated list of LOF variants reported so far is shown in ***Supplementary Table 1***(available online).

## ANGPTL3 and lipoprotein metabolism

ANGPTL3 is synthesized in the liver as a precursor protein, which is converted into the mature form by proteolytic cleavage by several hepatic pro-protein convertases (such as Furin, PCSK2, PCSK4, PACE4, PCSK5 and PCSK7). The cleavage process yields the active *N*-terminal fragment, which has efficient LPL inhibitory activity; this is supported by the observation that the deletion of ANGPTL3 amino terminal region causes the total loss of its inhibitory activity^[8^,^27]^. The cleavage process of ANGPTL3 appears to be facilitated by ANGPTL8. ANGPTL8 is secreted by the liver into the circulation where it interacts with ANGPTL3 for cleavage and forms a complex with the *N*-terminal fragment of ANGPTL3. The complex, as well as the free *N*-terminal fragment of ANGPTL3, inhibits LPL^[28]^. There is evidence in mice that ANGPTL8 has an LPL inhibitory motif, which is inactive as it is not accessible to the enzyme. The formation of the complex ANGPTL8-ANGPTL3 induces structural changes in ANGPTL8, which exposes the inhibitory motif for LPL inhibition. Thus, ANGPTL8 is inactive “per se” and requires ANGPTL3 to acquire LPL inhibitory activity. It has been demonstrated that the major ability of the ANGPTL8/ANGPTL3 complex to inhibit LPL depends on the active LPL inhibitory motif of ANGPTL8^[29]^. This is supported by the observations that the LPL inhibition by the ANGPTL3/ANGPTL8 complex could not be reversed by an anti-ANGPTL3 blocking antibody^[29]^.

Some genetic variants of ANGPTL8 affecting plasma lipids have been reported. Quagliarini *et al*. found that a common variant c.175C>T, p.Arg59Trp (rs2278426) was associated with lower plasma LDL-C and HDL-C levels in Hispanics and African Americans of the Dallas Heart Study^[28]^. In a genome wide association study (including mostly individuals of European descent), the p.Arg59Trp variant was found to be associated with a reduction of both LDL-C and HDL-C, but not to be associated with reduction of plasma triglycerides^[28]^. Furthermore, a low frequency variant of ANGPTL8 [c. 361C>T, p.Gln121* (rs145464906)] was identified in a survey of a large group of individuals by Peloso *et al*.^[30]^. Heterozygous carriers of this variant had higher HDL-C, lower TG and not significantly lower LDL-C levels as compared to non- carriers. It is conceivable to assume that the truncated ANGPTL8 generated by this variant is unable to form the ANGPTL8/ANGPTL3 complex, which is known to exert a strong inhibition on LPL activity^[31]^.

The mechanism of ANGPTL3-mediated inhibition of LPL is still not fully understood. Enzyme kinetic studies in cell free system showed that ANGPTL3 binds and reversibly inhibits the catalytic activity of LPL without affecting the LPL self-inactivation rate^[32]^. In the presence of cells, ANGPTL3 binds LPL attached to the cell surface, promotes the dissociation of full-length LPL from the cells and induces the cleavage of the enzyme by protease (PACE4 and Furin). This translates into the irreversible inactivation of the enzyme^[33]^.

In addition, ANGPTL3 has an inhibitory effect on the activity of the endothelial lipase (EL), an extracellular lipase, which carries mainly a phospholipase activity acting predominantly on HDL, and increases the catabolism of HDL particles. *Angptl3* deficient mice show low plasma levels of HDL-C, accompanied by increased phospholipase activity^[34]^.

**Fig.1:**
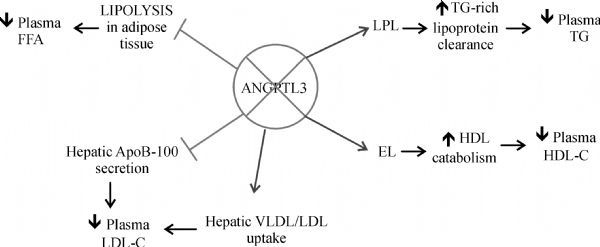
Effects of ANGPTL3 deficiency.

Treatment of mice with a monoclonal antibody against ANGPTL3 significantly reduced plasma HDL-C levels in wild-type but not in endothelial lipase deficient mice^[35]^ (***Fig. 1***).

## ANGPTL3 and metabolic conditions associated with hypertriglyceridemia

*Angptl3* gene expression and ANGPTL3 plasma levels may show marked changes in some metabolic disorders in humans or experimental manipulations in rodents which are characterized by marked elevation of the level of plasma triglycerides. These disorders include conditions such as diabetes, obesity, hypothyroidism, and leptin deficiency etc., as reported below.

### ANGPTL3 and diabetes

*In vitro* and *in vivo* studies have indicated that insulin acts as a negative regulator for ANGPTL3 production. In rat and human hepatoma cells, the amount of *Angptl3* mRNA and secreted ANGPTL3 protein decreased in a dose dependent fashion in the presence of insulin. In striking contrast, *Angptl3* gene expression and plasma protein level were increased in insulin-deficient streptozotocin-treated mice^[36]^. Inukai *et al*. confirmed that the level of *Angptl3* mRNA was increased in the liver of streptozotocin diabetic mice and this effect was reversed by administration of insulin^[37]^. In addition, the level of *Angptl3* mRNA and protein was increased more than 3 fold in type 2 diabetic obese mice (db/db mice)^[37]^.

Haridas *et al*. investigated the *in vivo* effect of insulin on circulating ANGPTL3 in humans^[38]^. They found that *in vivo* 6-hours euglycemic hyperinsulinemia decreased plasma ANGPTL3 (and ANGPTL8) at 3 and 6 hours. They also found that in immortalized human hepatocytes, insulin decreased *Angptl3* gene expression and ANGPTL3 secretion into the medium^[38]^. Therefore, also in humans, insulin decreases plasma ANGPTL3 by decreasing *Angptl3* expression in the liver. These combined results indicate that the expression of ANGPTL3 is increased in both insulin deficient and insulin resistant diabetic states, suggesting that increased plasma ANGPTL3 contributes to diabetic hypertriglyceridemia.

### ANGPTL3 and leptin deficiency

The role of leptin on the expression and plasma level of Angptl3 has emerged from early studies in mice. Shimamura *et al*. found that: (1) *Angptl3* mRNA expression and plasma Angptl3 levels were increased in both leptin resistant C57Bl/J6^*db/db*^ mice and in leptin deficient C57Bl/J6^*ob/ob*^ mice; (2) elevation of ANGPTL3 in plasma was associated with elevation of plasma TG; (3) leptin administration to leptin deficient *ob/ob* mice reversed *Angptl3* expression and ANGPTL3 plasma levels and induced a normalization of plasma TG^[36]^.

The role leptin on ANGPTL3 expression and plasma level in humans was recently documented in a study^[39]^ conducted in patients with generalized lipodystrophy, a leptin deficient condition associated with hypertriglyceridemia^[40]^. These patients were found to have increased plasma levels of ANGPTL3 (but not plasma levels of ANGPTL4), suggesting the possibility that hypertriglyceridemia might be the result of ANGPTL3 mediated inhibition of LPL^[39]^. After metreleptin treatment, the plasma level of leptin increased whereas the plasma level of ANGPTL3 and TG showed a significant decrease^[39]^. Thus, the finding of elevated ANGPTL3 levels in patients with lipodystrophy and their reduction following leptin treatment is consistent with the results obtained in leptin deficient mice and suggests that ANGPTL3 may contribute to hypertriglyceridemia in leptin deficient states.

### ANGPTL3 and hypothyroidism

Studies in humans with hypothyroidism and elevated plasma TG have shown that the administration of T4 induced a reduction of plasma VLDL cholesterol and triglyceride, associated with an increased LPL activity^[41]^. On the other hand, LPL activity has been found reduced in hypothyroidism and increased in hyperthyroid state^[41]^. Furthermore, the administration of selective agonists of thyroid hormone receptor beta (TRβ) resulted in a selective decrease of VLDL-TG in rodents^[42]^. The study by Fugier *et al*. showed that thyroid hormone down-regulates Angptl3 (but has no effect on Angptl4) in hypothyroid rats^[43]^. Using thyroid hormone receptor deficient mice, these investigators showed that thyroid hormone down-regulates *Angptl3* expression in a TRβ-dependent manner^[43]^. Therefore, decreased *Angptl3* expression and reduced ANGPTL3 secretion would result in increased LPL activity and a more rapid removal of plasma TG in hypothyroid patients after thyroid hormone administration.

### ANGPTL3 and LXR activation

LXR (Liver X Receptor) is a nuclear receptor that forms a heterodimer with RXR (retinoid X receptor) and activates the transcription of several genes involved in lipid metabolism. The compound T0901317 is a synthetic LXR ligand broadly used in the studies of LXR biology. Treatment of rodents with T0901317 causes an accumulation of TG in the liver and a marked increase of plasma TG^[44]^. While the accumulation of TG in the liver was explained by the increased expression of hepatic fatty acid synthase mediated by sterol regulatory element binding protein 1 (SREBP-1)^[44]^, the mechanism of hypertriglyceridemia has not been fully clarified. The involvement of Angptl3 in hypertriglyceridemia after LXR activation was firstly suggested by Inaba *et al*. who found that in human hepatoma cells LXR ligands induced an increased expression of *Angptl3* gene and an increased secretion of ANGPTL3 protein^[45]^. In addition T0901317 administration to C57BL/6J mice increased hepatic mRNA expression and plasma concentration of Angptl3, which were associated with a marked increase of plasma TG. By contrast, T0901317 administration to C57BL/6J-Angptl3 deficient mice (C57BL/6J *Angptl3*^hypl^, which do not produce Angptl3 mRNA or Angptl3 protein) increased hepatic triglyceride content but failed to increase plasma TG^[45]^. These results demonstrate that hypertriglyceridemia induced by LXR activation in mice was accounted for by LXR-mediated induction of *Angptl3* expression. Similar conclusions were reached by Kaplan *et al*. on the basis of *in vivo* as well as *in vitro* studies^[46]^.

### ANGPTL3 and acute phase reaction

The acute phase response is characterized by elevation of plasma TG due to both hepatic overproduction of VLDL and defect in the clearance of TG-rich lipoproteins, secondary to reduction of the LPL activity^[47]^. Treatment of mice with lipopolysaccharide (LPS) (a model of Gram-negative infection and an inducer of acute phase response) was found to induce a decreased expression of *Angptl3* in the liver and an increased expression of *Angptl4* in heart muscle and adipose tissue^[48]^. These results indicate that ANGPTL3 is not a positive acute phase protein and suggest that ANGPTL3 is not responsible for the reduction of LPL activity observed during acute phase response^[48]^.

## Mechanisms underlying the combined hypolipidemia in FHBL2

The increased LPL-mediated hydrolysis of TG in VLDL and chylomicrons explains the low levels of plasma TG in subjects with FHBL2. In addition to the enhanced clearance of TG-rich lipoproteins, the low level of circulating free fatty acid (FFA) present in mice and humans with *Angptl3* deficiency reduces the hepatic availability of FFA for the *de novo* synthesis of TG to be incorporated into VLDL^[13]^.

The catabolism of chylomicrons has been investigated in 7 homozygotes and 31 heterozygotes for the ANGPTL3 nonsense mutation p. (S17*)^[49]^. These subjects were investigated at fasting and at 6 hours after a fat rich meal. In homozygotes, the complete *Angptl3* deficiency was associated with a highly reduced postprandial hypertriglyceridemia, probably due to an accelerated catabolism of intestinal derived TG-rich lipoproteins (chylomicrons) secondary to the increased LPL activity. Additionally, heterozygotes with partial *Angptl3* deficiency displayed an attenuated postprandial lipemia, as compared to controls^[49]^.

The mechanism for low LDL-C levels in *Angptl3* deficiency is a matter of active investigation. In* in vivo *studies of lipoprotein metabolism in carriers of *Angptl3* LOF variant showed a reduced VLDL-apoB (the main protein constituent of VLDL) production rate and an increased LDL-apoB fractional catabolic rate, with a gene-dosage effect, suggesting that ANGPTL3 regulates hepatic lipoprotein secretion and clearance^[11]^.

Wang *et al*. reported that in mice with genetic deficiencies in key proteins involved in lipoprotein clearance (e.g. apoe^-/-^ or ldlr^-/-^ mice), the inactivation of ANGPTL3 with an ANGPTL3 monoclonal antibody reduced the hepatic production of VLDL-TG but not that of VLDL-apoB^[50]^. The decrease in hepatic TG secretion in *Angptl3* deficient mice is caused by a decreased supply of FFA from the circulation into the liver for hepatic *de novo* synthesis of TG. Shortage of TG is expected to decrease VLDL lipidation^[50]^. This finding is in keeping with the observation that carriers of *Angptl3* LOF variants and *Angptl3* deficient mice have low plasma FFA levels due to the absence of ANGPTL3-stimulated lipolysis in adipose tissue^[13]^.

Alternatively, the low LDL plasma levels may be the result of receptor-mediated catabolism of LDL. However, the observation that *Angptl3* deficient mice lacking functional proteins involved in LDL plasma clearance (e.g. the ldlr^-/-^ or apoe^-/-^ mice) had a reduction of plasma LDL-C similar to that of wild-type *Angptl3* deficient mice, indicated that the reduction of LDL-C in ANGPTL3 deficiency is independent of the canonical receptor-mediated clearance pathways^[50^–^51]^. It was suggested that Angptl3 inactivation in mice increases the clearance of VLDL remnants (which are largely converted to LDL in the circulation), leading eventually to a reduced plasma level of LDL.

Recently, Xu *et al*. performed RNAi-mediated *Angptl3* gene silencing in five mouse models and in human hepatoma cells and validated the results by deleting *Angptl3* gene *in vitro* using CRISPR/Cas9 genome editing^[52]^. They found that hepatic *Angptl3* silencing in multiple mouse models is sufficient to reduce plasma LDL-C levels. On the other hand, in human hepatoma cells, *Angptl3* silencing and deletion reduced apoB secretion and increased LDL/VLDL uptake^[52]^. These results are consistent with the *in vivo* turnover study conducted by Musurunu *et al*. in subjects with familial combined hypolipidemia, as mentioned above^[11]^.

The low levels of plasma HDL-C present in FHBL2 subjects may be the result of the increased activity of endothelial lipase (EL), as found in mice^[34]^. The reduction of plasma HDL-C was found to be associated with qualitative changes of HDL, which have also a reduced size^[14^,^19]^. In addition, it was reported that the function of the HDL in FHBL2 is impaired, as plasma of subjects with complete deficiency of ANGPTL3 showed a reduced cell cholesterol efflux capacity through various efflux pathways^[14]^.

## Plasma levels of ANGPTL3

*In vivo,* ANGPTL3 is cleaved by proprotein convertases to yield the biologically active *N*-terminal fragment and an inactive *C*-terminal fragment. Thus, the forms of ANGPTL3 present in plasma are the full length as well as the *N*-terminal and *C*-terminal fragments. To measure the plasma level of ANGPTL3 two quantitative sandwich ELISA assays have been developed. The first method used commercially available rabbit polyclonal anti-human ANGPTL3 antibody and biotin labelled anti-human ANGPTL3 rabbit polyclonal antibody as a capture and detection antibody respectively (Biovendor assay). This method is likely to detect the full-length ANGPTL3 protein as well as the cleaved forms of the protein depending on the epitope localization^[53]^. The second method employed a rabbit polyclonal antibody raised against the N-terminal recombinant human ANGPTL3 as a capture antibody and a biotinylated sheep IgG raised against human full-length recombinant ANGPTL3 as detection antibody. This method should detect the full-length protein but does not distinguish the two cleaved forms^[54]^.

The concentration of ANGPTL3 in plasma of healthy subjects is highly variable, probably depending on the type of antibodies used and the different populations investigated. The mean plasma level of ANGPTL3 was found to be (470±122) ng/mL^[35]^ and (764±291) ng/mL^[55]^ in the Japanese population while in the Finnish population the mean concentration was found to be (368±168) ng/mL^[54]^. The plasma level of ANGPTL3 has shown a positive correlation with plasma HDL-C and LDL-C^[54^–^56]^. The correlation between plasma levels of ANGPTL3 and TG is still controversial; in one study a negative correlation was found^[54]^ whereas in another study such a correlation was not observed^[56]^. In subjects homozygous or compound heterozygous for LOF mutations in ANGPTL3, plasma ANGPTL3 was undetectable while in simple heterozygotes its level was reduced by 50%–60% of the values found in the control subjects^[13^,^49^,^57]^.

## ANGPTL3 as therapeutic target

The marked reduction of the level of atherogenic lipoproteins (VLDL and LDL) observed in FHBL2 subjects suggested that the inactivation of ANGPTL3 may be used as a therapeutic strategy in the management of dyslipidemic conditions. Two different approaches have been used to inactivate ANGPTL3. The first is based on the administration of a human monoclonal antibody against ANGPTL3. When administered to monkeys and dyslipidemic mice, this antibody induced a marked reduction of plasma TG, LDL-C and HDL-C^[35]^. The treatment of healthy human dyslipidemic volunteers with ANGPTL3-blocking antibody (evinacumab) was found to reduce TG and LDL-C by 76% and 23%, respectively. In dyslipidemic mice, evinacumab was found to reduce total cholesterol and TG levels by 50% and 85% respectively, as well as the size of atherosclerotic plaques and their necrotic content, providing a proof of principle that the combined hypolipidemia associated with therapeutic inhibition of ANGPTL3 is anti-atherogenic^[25]^. Evinacumab was also administered to nine patients with homozygous familial hypercholesterolemia due to complete deficiency of LDLR. Four-week treatment reduced LDL-C, TG and HDL-C by approximately 50%, 47% and 36%, respectively, in addition to reductions in baseline levels already achieved with aggressive lipid-lowering therapy^[58]^.

The second approach is based on the administration of antisense oligonucleotides (ASOs) targeting hepatic *Angptl3* mRNA, which is expected to markedly reduce the level of ANGPTL3 protein. ASO treatment in mice reduced the levels of TG and LDL-C as well as liver TG content and retarded the progression of atherosclerosis. In Phase 1 trial in humans, treatment for 6 weeks with multiple doses reduced the plasma concentrations of TG by a maximum of 63%, LDL-C by a maximum of 32.9%, with no significant changes in HDL-C and without important side effects^[59]^.

## Conclusions

The discovery of ANGPTL3 deficiency in mice and humans has stimulated a series of studies which have clarified the role of ANGPTL3 in lipoprotein metabolism. These investigations have suggested that ANGPTL3 may be a novel therapeutic target in the management of dyslipidemias in humans. The results of recent intervention trials aimed at inhibiting ANGPTL3 appear to support this hypothesis.
